# Stage-dependent changes in culture medium osmolality promote porcine oocyte maturation *in vitro*

**DOI:** 10.3389/fcell.2025.1524749

**Published:** 2025-01-30

**Authors:** Lixiang Liu, Yu Tang, Jing Shao, Bingfeng Fan, Yifeng Yang, Ying Zhang, Xiangyuan Zhao, Hailong Xue, Huimin Sun, Xulin Zhang, Yushi Zhang, Baozeng Xu

**Affiliations:** ^1^ Institute of Special Animal and Plant Sciences, Chinese Academy of Agricultural Sciences, Changchun, Jilin, China; ^2^ State Key Laboratory for Molecular Biology of Special Economic Animals, Institute of Special Animal and Plant Sciences, Chinese Academy of Agricultural Sciences, Changchun, Jilin, China

**Keywords:** porcine oocytes, cell volume regulation, osmolarity, glycine, *in vitro* maturation

## Abstract

**Introduction:**

Early preimplantation embryos of mammals exhibit pronounced sensitivity to hyperosmotic conditions, which results in an embryonic developmental block. The reduction of medium osmolarity or the supplementation with organic osmolytes can eliminate this arrest. Therefore, cell volume homeostasis is essential for embryonic development *in vitro*. Oocytes become capable of independent volume regulation after detaching from the follicle microenvironment. Whether the efficiency and quality of oocyte maturation can be improved by optimizing cell volume regulation by adjusting the osmolality of the culture medium in the presence of the organic osmolyte of glycine remains to be determined.

**Methods:**

The IVM of porcine oocytes was divided into two stages, i.e. the first 22 h as the first stage, and the last 22 h as the second stage. In the presence of 1 mM glycine, we adjusted the osmolality of the culture medium from low to high (290 mOsM for the first 22 h and 320 mOsM thereafter) by adding raffinose, which cannot be used by animal cells, in a culture stage-dependent manner.

**Results:**

Stage-dependent adjustment of simplified medium PZM-3 osmolarity (290 mOsM for the first 22 h and 320 mOsM thereafter) in the presence of 1 mM glycine significantly improved the quality of porcine oocyte maturation *in vitro*, manifested by the oocyte maturation rate, functional mitochondrial distribution and activity, the transcript levels of glycolysis genes in granulosa cells, and subsequent embryonic developmental ability and ROS levels.

**Conclusion:**

Our study demonstrates that optimizing cell volume regulation can further enhance the developmental potential of oocytes cultured *in vitro*.

## Introduction

*In vitro* maturation (IVM) of mammalian oocytes facilitates embryo generation via *in vitro* fertilization, provides oocytes for somatic cell nuclear transfer, and derives haploid stem cells from gametes (Lonergan and Fair, 2016). Despite advances in this technology, IVM efficiency in pigs is significantly lower than in species such as mice, cattle, and sheep. This difference has profound implications for the application of cutting-edge reproductive biotechnologies and may hinder the improvement of reproductive performance in high-quality sows and the establishment of porcine models of human diseases. Therefore, optimization of porcine oocyte IVM has become a key area of research in the global porcine reproductive community. In addition to hormone levels (Lu et al., 2014; Sakaguchi and Nagano, 2020), amino acid availability (Bahrami et al., 2019; Lee et al., 2019), and antioxidant supplementation (Das et al., 2014; Li et al., 2019; Cajas et al., 2020), maintenance of cell volume homeostasis has been identified as an important determinant of oocyte maturation quality (Baltz and Zhou, 2012).

More than a century of experience in mammalian embryo culture has highlighted the pivotal role of cell volume control in determining the developmental trajectory of preimplantation embryos (Biggers, 1998). Early endeavors to cultivate mammalian embryos were predicated on biomimicry, positioning fertilized ova in media with osmolarities approximating those of the organism’s internal milieu (290–310 mOsM). This approach, however, resulted in species-specific embryonic arrest, attributed to the osmotic conditions (Goddard and Pratt, 1983; Camous et al., 1984; Bolton et al., 1989; Kishi et al., 1991). Notably, culture media that successfully overcome this developmental block either reduce the medium’s osmolarity or incorporate organic osmolytes such as glycine (Gly), betaine, β-alanine, and glutamine into a medium with an osmolarity of ∼310 mOsM (Van Winkle et al., 1990; Biggers et al., 1993). For instance, culturing mouse embryos in KSOM or CZB media with reduced osmolarity (250–275 mOsM) has been shown to surmount the two-cell arrest (Chatot et al., 1990; Lawitts and Biggers, 1991; 1993; Hadi et al., 2005). The rapid restoration of cell volume control, when disturbed by external conditions, is mediated by the activation of the Na^+^/H^+^ exchanger NHE1 and the HCO_3_^+^/Cl^−^ exchanger AE2, which adjusts intracellular concentrations of Na^+^ and Cl^−^. Nonetheless, it is crucial to avoid excessively high ionic concentrations that could disrupt normal cellular physiological and biochemical processes. Subsequently, preimplantation embryos and oocytes reactivate specific organic osmolyte channels to internalize uncharged osmolytes, replacing inorganic ions and ensuring that cells maintain normal physiological and biochemical processes (Alper, 2009; Donowitz et al., 2013; Nakajima et al., 2013; Tscherner et al., 2021). Research into the cellular volume regulatory mechanisms during mouse oocyte meiosis has indicated that specific deletion of Slc6a9 encoding the Gly transporter eliminates Gly transport in preimplantation embryos and their ability to counter hypertonic stress (Tscherner et al., 2023). These findings underscore the necessity of precise cellular volume regulation for the healthful development of mammalian oocytes and preimplantation embryos.

Gly is an indispensable precursor in the synthesis of proteins and nucleic acids, which is essential for rapid cellular proliferation (Redel et al., 2016; Alves et al., 2019). It has been reported that Gly is the most abundant amino acid in the porcine follicular fluid (Hong and Lee, 2007), which suggests that Gly may be an important factor to be considered for the improvement of oocyte maturation *in vitro*. While the precise mechanism remains to be fully elucidated, emerging evidence points to a significant role for Gly as an organic osmolyte in the development of bovine embryos and mouse oocytes (Zhou et al., 2013; Herrick et al., 2016; Anchordoquy et al., 2019; Zuo et al., 2020). Additionally, fluctuations in the uterine fluid osmolarity of estrous sows, with an increase of 9.2% from day 3 to day 5 (Li et al., 2007), reflect the dynamic nature of the physiological osmotic environment within the mammalian reproductive system. Our previous findings have indicated that the addition of Gly during the vitrification/thawing of porcine oocytes mitigates osmotic damage and enhances developmental competence (Tang et al., 2022). This has led to the hypothesis that porcine oocytes can adapt to changes in the culture medium osmolarity during *in vitro* maturation and utilize Gly to regulate cell volume homeostasis, potentially improving the quality of oocyte maturation and the ensuing embryonic development.

In this study, we used the simple medium PZM-3 as the basic medium and adjusted the osmolality of the culture medium from low to high (290 mOsM for the first 22 h and 320 mOsM thereafter) by adding raffinose, which cannot be used by animal cells, in a culture stage-dependent manner and supplemented with 1 mM glycine. This strategy significantly improved the quality of porcine oocyte maturation *in vitro*, manifested by the oocyte maturation rate, functional mitochondrial distribution and activity, the transcription level of granulosa cell glycolysis genes, and subsequent embryonic developmental ability and ROS levels. Our study demonstrates that optimizing cell volume regulation can further enhance the developmental potential of oocytes cultured *in vitro*.

## Materials and methods

### Reagents

Unless otherwise noted, all chemicals and reagents used in this study were purchased from Sigma Chemical Company (St. Louis, MO, United States).

### Oocyte collection and *in vitro* maturation

Porcine ovaries were collected from a local slaughterhouse and transported to the laboratory within a 2-hour window in a saline solution containing antibiotics (100 IU/mL penicillin and 0.1 mg/mL streptomycin sulfate). Follicular fluid was extracted from follicles, 3–7 mm in diameter, and connected to a 10 mL syringe using an 18-gauge needle. After a 15-minute settling period, cumulus-oocyte complexes (COCs) exhibiting at least three layers of cumulus cells and homogeneous cytoplasm were selected under a stereomicroscope for further experimentation. A group of 50 COCs was cultured in 500 μL of MI maturation medium, which was a composite of M199 (Gibco, Grand Island, NY, United States) or PZM-3 supplemented with 3.05 mM glucose, 0.57 mM cysteine, 0.91 mM sodium pyruvate, 0.15 mM kanamycin, 0.1% (w/v) PVA, 10% porcine follicular fluid (PFF), 10 ng/mL epidermal growth factor (EGF), 0.5 μg/mL luteinizing hormone (LH), 0.5 μg/mL follicle-stimulating hormone (FSH), and 100 IU/mL insulin transferrin selenium (ITS). This culture was maintained for 22 h, after which the oocytes were transferred to MII medium, which is MI medium devoid of LH and FSH, and cultured for an additional 22 h. The incubator conditions were set at 38.5°C, 5% CO_2_, and 5% humidity.

### Parthenogenetic activation

Following IVM, CCs were removed by repeated aspiration in a maturation medium containing 300 IU/mL hyaluronidase. Oocytes that displayed the first polar body (MII oocytes) were selected for parthenogenetic activation (PA) using an activation solution composed of 0.26 M mannitol, 0.1 mM MgCl_2_, 0.1 mM CaCl_2_, 0.5 mM HEPES, and 0.05% BSA. The activation was performed using a DC pulse of 150 V/mm, 60 μs, applied twice. Activated oocytes were then cultured in PZM-3 medium containing 5 μg/mL cytochalasin B (CB) for 4 h to prevent the extrusion of the second polar body, after which they were transferred to PZM-3 medium without CB for continued culture. Developmental progress was monitored by recording cleavage and blastocyst rates at 48 and 168 h, respectively.

### *In vitro* fertilization

Ten to fifteen denuded MII oocytes were washed in modified Tris Buffered Medium (mTBM) and placed in 60 μL drops of mTBM for IVF. The mTBM contained 113.1 mM NaCl, 3.0 mM KCl, 7.5 mM CaCl_2_·2H_2_O, 20.0 mM Tris Base, 11.0 mM glucose, 5.0 mM sodium pyruvate, 1.0 mM caffeine, and 0.2% BSA. Porcine spermatozoa, cryopreserved in capillaries, were thawed in a 37°C water bath for 1 min and centrifuged at 700 g for 3 min in mTBM, then washed twice. The sperm pellet was resuspended in 1 mL pre-warmed (38.5°C) mTBM and incubated for 15 min at 38.5°C. The MII oocytes were fertilized in mTBM with a sperm concentration of 1 × 10^5^ sperm/mL for 6 h, after which they were stripped of sperm attached to the zona pellucida using a fine glass pipette. Groups of ten fertilized ova were cultured in each PZM-3 drop under paraffin oil, and developmental rates, including cleavage and blastocyst formation, were assessed at 48 and 168 h, respectively.

### Mitochondrial staining

MitoTracker™ Red CMXRos (ThermoFisher, M7512, United States) was employed as a mitochondrial probe, used according to the manufacturer’s protocol. Denuded MII oocytes were incubated in a solution of M199/PZM-3 with 0.1% PVA and 500 nM Mitotracker™ Red for 30 min at 38.5°C, 5% CO_2_, and full humidity. After three washes in PBS containing 0.1% PVA, the oocytes were fixed with 4% PFA for 30 min and then mounted for observation using a laser confocal scanning microscope (Nikon C2, Japan).

### Detection of cortical granule distribution

Denuded MII oocytes were treated with 1% HCl for 20 s at room temperature to remove the zona pellucida. The zona-free oocytes were fixed with 4% PFA for 30 min at room temperature, then permeabilized with 2% Triton X-100 in PBS at 37°C overnight, and blocked with 3% BSA for 1 h. The oocytes were incubated with 100 μg/mL LCA-FITC (ThermoFisher, L32475, United States) for 30 min at room temperature in a humidified dark chamber. After washing three times in PBS containing 0.1% PVA, oocytes were placed on glass slides and examined using confocal laser scanning microscopy.

### ROS level detection

The level of ROS in oocytes was measured using a ROS assay kit (Biyuntian, S0033S, China). Oocytes devoid of CCs were incubated in culture medium with 0.1% PVA and 10 μM DCFH-DA for 20 min under incubator conditions. After washing three times in PBS containing 0.1% PVA, oocytes were placed on glass slides and examined using confocal laser scanning microscopy.

### Quantitative real-time PCR

Total RNA was extracted from CCs of 30 COCs using the RNeasy Mini Kit (Qiagen, Hilden, Germany), following the manufacturer’s guidelines. RNA concentration and purity were assessed using a spectrophotometer (DeNovix Inc., Wilmington, Delaware, United States). The RNA was reverse transcribed into cDNA using the RevertAid First Strand cDNA synthesis kit (Thermo Scientific), and the resulting cDNA was used for qRT-PCR analysis or stored at −80°C. qRT-PCR was conducted following the protocol of the FastStart Universal SYBR Green Master kit (Roche Applied Science, Penzberg, Germany). Primer details are presented in Table 1. The GAPDH gene served as an endogenous control, and relative quantification of mRNA transcript levels was performed using the 2^−ΔΔCt^ method.

**TABLE 1:** Primers used for RT-PCR analyses.

Gene	Forward primer sequence (5′-3′)	Reverse primer sequence (5′-3′)	Product size (bp)	GenBank accession number
GAPDH	AAG​TTC​CAC​GGC​ACA​GTC​AA	CAG​CAT​CGC​CCC​ATT​TGA​TG	109	KJ786424.1
Ldh1	ACA​CTG​GAA​AGC​GGT​TCA​CA	TTC​TGC​CAG​ATC​TGC​CAC​AG	109	NM_001172363.2
Pkfp	GGA​GTT​CTG​TGT​CCC​CAT​GG	TGT​CGG​TGA​TGG​TGT​TGA​GG	107	XM_021065066.1
Pkm2	TCA​TTC​AGA​CCC​AGC​AGC​TG	TGG​TAC​AGA​TGA​TGC​CGG​TG	117	CV864390.1

### Measurement of ATP contents

ATP contents in oocytes were determined by using the assay kit (Bioluminescent Somatic Cell Assay Kit, Catalog #FLASC, St Louis, MO) based on the luciferin–luciferase reaction as previously described with minor modifications (Van Blerkom et al., 1995; Combelles and Albertini, 2003). Briefly, 20 denuded MII oocytes or CCs derived from 20 to 30 COCs in a group were snap-frozen in microcentrifuge tubes containing 160 μL and 180 μL water, respectively, and then stored at −80°C. For ATP assays, 50 μL of each thawed sample or standard solution with known ATP content was added to 100 μL ice-cold Cell ATP-Releasing Reagent, incubated on ice for 5 min, and then 100 μL ice-cold ATP Assay Mix (diluted 1:25 in assay mix buffer) was added. The reaction mixture was then incubated for 10 min at room temperature in the dark. Bioluminescence of each sample and 10 different standards with known ATP content (0–90 pmol ATP) was measured using a high-sensitivity luminometer. In each measurement, samples and standards were analyzed three times, and the average value was used to express the test results. Finally, the ATP content in each sample was calculated based on the 10-point standard curve for each measurement. The DNA concentration was determined in the remaining CCs lysate samples and used to normalize the ATP content in each sample. ATP contents in oocytes were expressed as pmol/oocyte.

### Statistical analysis

Plots were generated and statistical analyses were performed using GraphPad Prism 6 (GraphPad Software, Inc., San Diego, CA). Fluorescence intensity was quantified with ImageJ software (National Institutes of Health, United States). All experiments were performed independently at least three times. Data expressed in proportions were analyzed after arcsine transformation. Data are presented as the mean ± standard error of the mean (SEM) and were analyzed using one-way ANOVA with Tukey’s multiple comparisons test. Different low case letters indicate statistical differences at *P* < 0.05.

## Results

### Regulation of culture medium osmolarity and addition of Gly significantly enhance porcine oocyte maturation rates

Our preliminary data (see Supplementary Figure 1) suggest that media osmolarity and organic osmolytes significantly influence both oocyte maturation rates and cleavage rates *in vitro*. Based on this, we set up the following four groups to further investigate the quality of oocyte maturation in various maturation media.① M199, ② PZM-3 (290 mOsM), ③ PZM-3↑ (290 mOsM to 320 mOsM), ④ PZM-3↑ (290 mOsM to 320 mOsM) + Gly.

**FIGURE 1:**
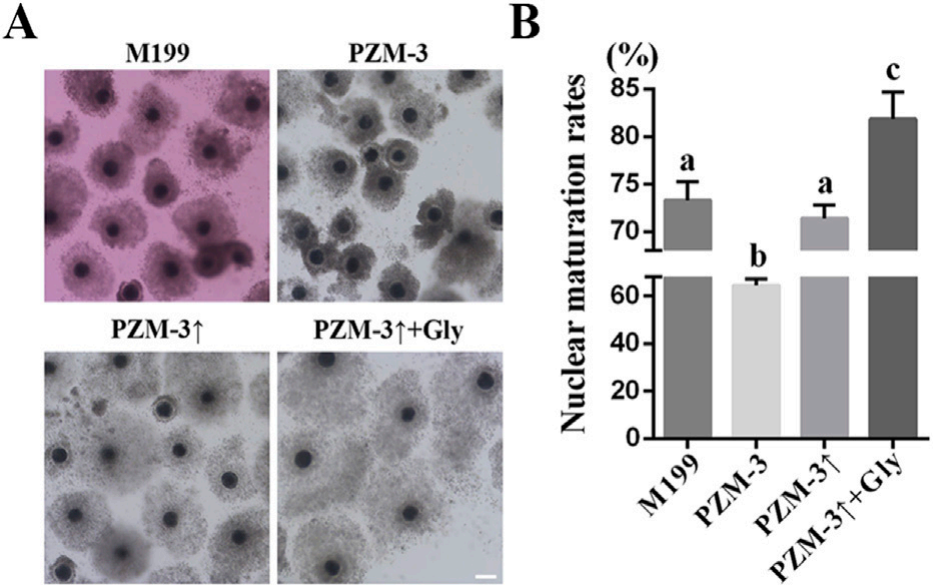
Effects of different culture mediums on porcine oocyte maturation rates. **(A)** Photomicrographs of representative COCs following a 44 h maturation (scale bar: 200 μm). **(B)** Nuclear maturation rate, defined as the proportion of oocytes with the first polar body (number of MII oocytes/number of viable oocytes). All experiments were performed independently at least three times. Data expressed in proportions were analyzed after arcsine transformation. Data are presented as the mean ± SEM. Different lowcase letters indicate statistical differences at *P* < 0.05. M199: Oocytes were matured in a M199-based medium for 44 h *in vitro*. PZM-3: Oocytes were matured in a PZM-3-based medium for 44 h *in vitro*. PZM-3↑: Oocytes were cultured in a PZM-3-based medium initially adjusted to an osmolarity of 290 mOsM for the first 22 h, then incremented to 320 mOsM for the remaining 22 h. PZM-3↑ + Gly: Oocytes were matured in a PZM-3 medium supplemented with 1 mM Gly, with osmolarity adjusted as described for PZM-3↑.

After 44 h of *in vitro* culture, the oocyte maturation rates were assessed. The PZM-3 group exhibited a markedly lower maturation rate (64.48% ± 2.34%, n = 125) compared to the M199 group (73.30% ± 1.71%, n = 122). 71.37% ± 1.25% (n = 135) of oocytes reached to the MII stage was observed in the PZM-3↑ group, which did not differ significantly from the M199 group. Notably, the PZM-3↑ + Gly group achieved the highest maturation rate of 81.88% ± 2.42% (n = 124) (Figure 1). These findings indicate that optimizing cell volume regulation through adjusting the osmolarity of the PZM-3 medium from 290 mOsM during the first 22 h to 320 mOsM during the second 22 h in the presence of Gly can substantially augment the IVM efficiency of porcine oocytes compared to the conventional M199 medium.

### Optimizing the cell volume regulation of porcine oocytes during meiosis improves the subsequent embryonic development

**FIGURE 2:**
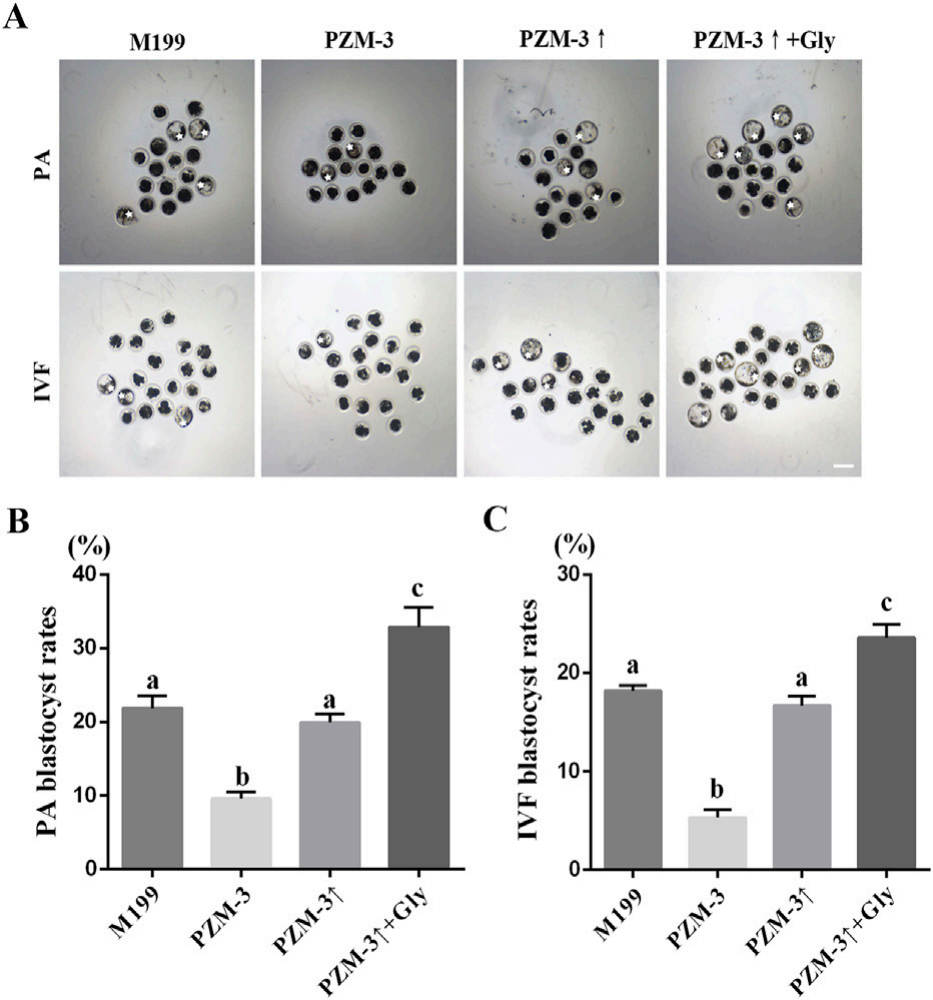
Effects of different culture mediums on embryonic development post-maturation in porcine oocytes. **(A)** Photomicrographs illustrating the morphological progression of embryos derived from PA and IVF up to the seventh day of development (scale bar: 200 μm). **(B)** The rate of blastocyst formation following PA is calculated as the number of blastocysts formed divided by the total number of oocytes activated. **(C)** The rate of blastocyst formation after IVF is calculated as the number of blastocysts formed divided by the total number of oocytes fertilized. All experiments were performed independently at least three times. Data expressed in proportions were analyzed after arcsine transformation. Data are presented as the mean ± SEM. Different low case letters indicate statistical differences at *P* < 0.05.

The maturation quality oocyte is a determinant of embryonic development (Gilchrist et al., 2024). To further investigate the quality of oocyte maturation in various maturation medium, we evaluated the developmental potential of embryos generated either by parthenogenetic activation (PA) or *in vitro* fertilization (IVF) of oocytes matured in different media. The 2-cell embryos from PA in the PZM-3 group were significantly decreased compared with those in the M199 group; there was no significant difference between the PZM-3↑group and the M199 group, and the 2-cell embryos in the PZM-3↑ + Gly group were significantly higher than those in the M199 group. The 4-cell embryo and subsequent developmental stages up to the blastocyst also showed a similar trend. The specific data are shown in Table 2 and Figure 2.

**TABLE 2:** Development of PA embryos.

Groups	No. embryos cultured	2-cell rate (%)	4-cell rate (%)	8-16cell rate (%)	Morula%	Blastocyst (%)
M199	64	78.30 ± 2.47^a^	69.20 ± 4.83^a^	51.87 ± 4.65^ab^	40.82 ± 2.37^ab^	21.90 ± 1.35^a^
PZM-3	63	57.49 ± 4.49^b^	43.42 ± 7.30^b^	30.64 ± 6.45^c^	22.51 ± 3.76^c^	9.58 ± 0.75^b^
PZM-3↑	55	76.23 ± 2.72^a^	62.21 ± 4.70^a^	45.37 ± 1.50^b^	31.36 ± 5.11^b^	19.93 ± 0.94^a^
PZM-3↑ + Gly	55	87.37 ± 1.70^c^	72.60 ± 1.82^a^	61.83 ± 3.45^a^	49.31 ± 3.28^a^	32.88 ± 2.19^c^

Note: Data are presented as the mean ± SEM. Data expressed in proportions were analyzed after arcsine transformation. Different lowcase letters indicate statistical differences at *P* < 0.05.

The 2-cell embryos generated from IVF in the PZM-3 group were significantly less than those in the M199 group, and there was no significant difference between the PZM-3↑ group and the M199 group. The 2-cell embryos in the PZM-3↑ + Gly group were significantly higher than that in the M199 group. The 4-cell embryos and other embryos showed a similar trend. The specific data are shown in Table 3 and Figure 2. Collectively, these results suggest that optimizing cell volume regulation during oocyte maturation can significantly bolster the subsequent embryonic developmental competence.

**TABLE 3:** Development of IVF embryo.

Groups	No. embryos cultured	2-cell rate (%)	4-cell rate (%)	8-16cell rate (%)	Morula%	Blastocyst (%)
M199	55	72.77 ± 1.80^a^	58.62 ± 3.25^a^	41.95 ± 1.28^a^	29.31 ± 1.63^ab^	18.19 ± 0.45^a^
PZM-3	57	59.88 ± 2.19^b^	45.46 ± 1.61^b^	31.11 ± 4.37^b^	17.18 ± 3.33^c^	5.34 ± 0.65^b^
PZM-3↑	54	72.37 ± 3.29^a^	55.56 ± 3.93^a^	40.72 ± 1.35^a^	25.87 ± 1.76^b^	16.70 ± 0.76^a^
PZM-3↑ + Gly	51	84.40 ± 2.23^c^	59.07 ± 6.48^a^	49.13 ± 3.52^c^	35.14 ± 3.12^a^	23.58 ± 1.13^c^

Note: Data are presented as the mean ± SEM. Data expressed in proportions were analyzed after arcsine transformation. Different lowcase letters indicate statistical differences at *P* < 0.05.

### Optimizing cell volume regulation during oocyte maturation significantly enhance the clustered mitochondrial distribution in porcine oocytes

**FIGURE 3:**
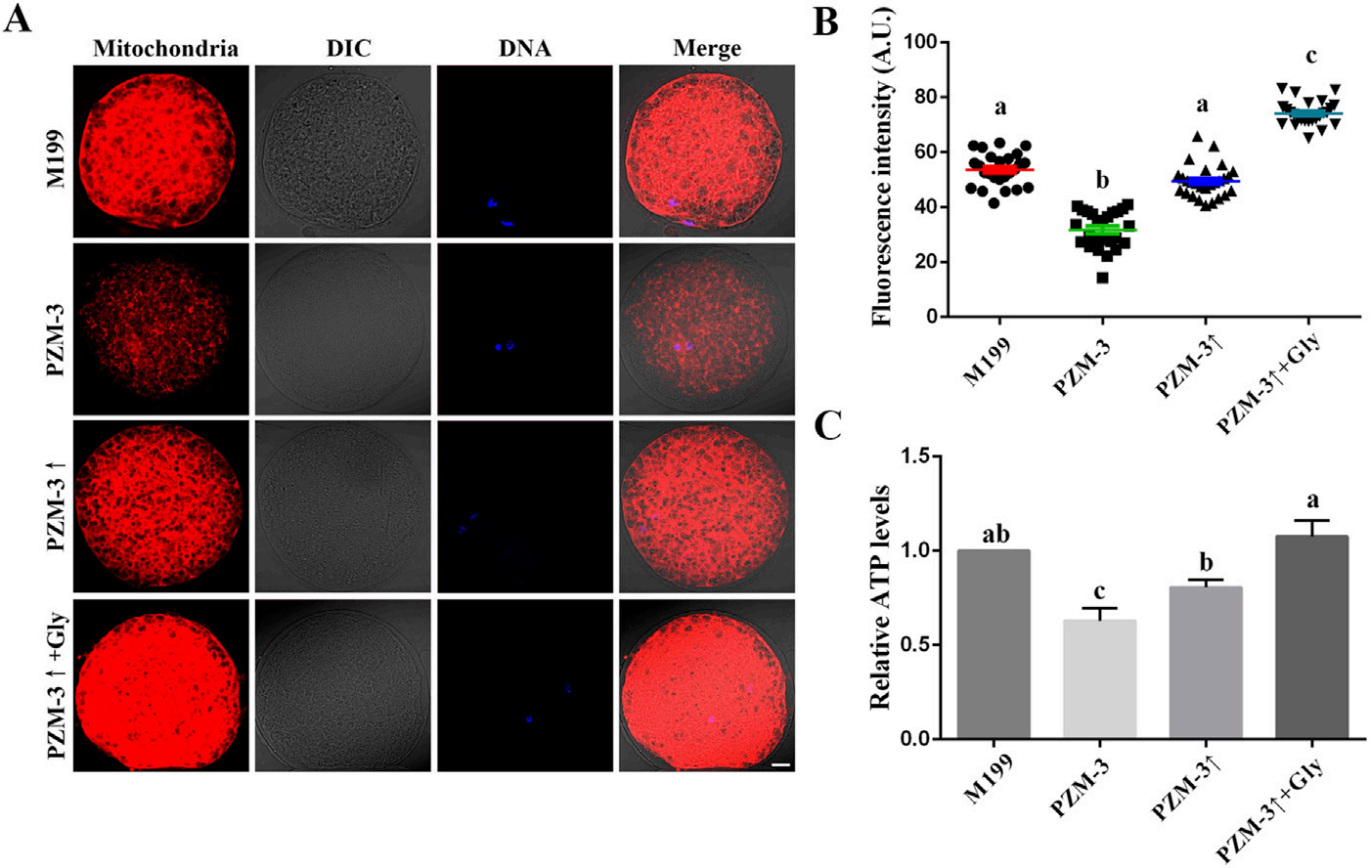
Effects of different culture mediums on mitochondrial localization, activity, and ATP content in porcine oocytes during maturation. **(A)** Immunofluorescent visualization of mitochondrial distribution within oocytes, depicted in red. The images include Differential Interference Contrast (DIC) microscopy to provide cellular context; DAPI nuclear staining is shown in blue; and a merged image represents the overlay of DIC, mitochondrial red, and nuclear blue channels (scale bar: 10 μm). **(B)** Graphical representation of the quantified mitochondrial fluorescence intensity, comparing across oocytes from the distinct experimental groups, indicating the relative mitochondrial activity. **(C)** Comparative assessment of ATP content within oocytes, as determined for each group, reflecting the bioenergetic status of the cells during the maturation phase. All experiments were performed independently at least three times. Data are presented as the mean ± SEM. Different low case letters indicate statistical differences at *P* < 0.05.

Oocyte meiotic maturation refers to the coordinated and synchronous maturation of the nucleus and cytoplasm. Mitochondrial activity and distribution are two of the criteria for measuring cytoplasm maturation (Chen et al., 2021). The mitochondrial distribution pattern of pig oocytes was characterized by two main features: one was distributed evenly throughout the cytoplasm (homogeneous), and the other was distributed unevenly within the cytoplasm (heterogeneous). These two main groups of mitochondrial distribution were divided further concerning the aggregation of mitochondria: small pixels of fluorescence intensity—fine; small linear spots of fluorescence intensity—crystalline; bigger areas of fluorescence with irregular shapes—granulated; and aggregates of bigger fluorescent areas—cluster. The fine aggregation type was found in homogeneously distributed oocytes only; the cluster aggregation type only in the heterogeneously distributed oocytes (Torner et al., 2004). The PZM-3↑ + Gly group displayed a significant increase in cluster distribution (57.72% ± 1.72%, n = 104) compared to the M199 group (41.16% ± 2.57%, n = 93). The PZM-3↑group’s mitochondrial distribution was comparable to the M199 group, whereas the PZM-3 group showed a significant decrease in cluster distribution (12.80% ± 3.10%, n = 98) (Table 4). Mitochondrial activity, as indicated by fluorescence intensity per oocyte, was lowest in the PZM-3 group (31.74 ± 6.72, n = 25) and increased significantly in the PZM-3↑ + Gly group (74.15 ± 4.35, n = 25) compared to the M199 group (53.64 ± 5.6, n = 25) (Figures 3A, B). The ATP content in the PZM-3 group was significantly lower than that in the M199 group, while the ATP content in the PZM-3↑ + Gly group was significantly higher than that in the PZM-3↑ group, but no significance from that in the M199 group (Figure 3C). This suggests that optimizing cell volume regulation during oocyte maturation significantly enhance the clustered mitochondrial distribution in porcine oocytes.

**TABLE 4:** Statistics of mitochondrial distribution patterns in the MII oocytes.

Groups	Number of oocytes	Mitochondrial distribution (%)					
		Homogeneous			Heterogeneous		
		Fine	Crystalline	Granulated	Crystlline	Granulated	Cluster
M199	93	14.82 ± 2.64^a^	0	0	0	44.02 ± 1.31^a^	41.16 ± 2.57^a^
PZM-3	98	37.67 ± 4.20^b^	0	0	0	49.21 ± 1.57^a^	12.80 ± 3.10^b^
PZM-3↑	105	13.37 ± 1.42^a^	0	0	0	48.40 ± 2.42^a^	38.61 ± 1.68^a^
PZM-3↑ + Gly	104	14.42 ± 2.31^a^	0	0	0	28.37 ± 3.61^b^	57.72 ± 1.72^c^

Note: Data are presented as the mean ± SEM. Data expressed in proportions were analyzed after arcsine transformation. Different lowcase letters indicate statistical differences at *P* < 0.05.

### Reduction of ROS levels in porcine oocytes through regulating culture medium osmolarity in the presence of Gly

**FIGURE 4:**
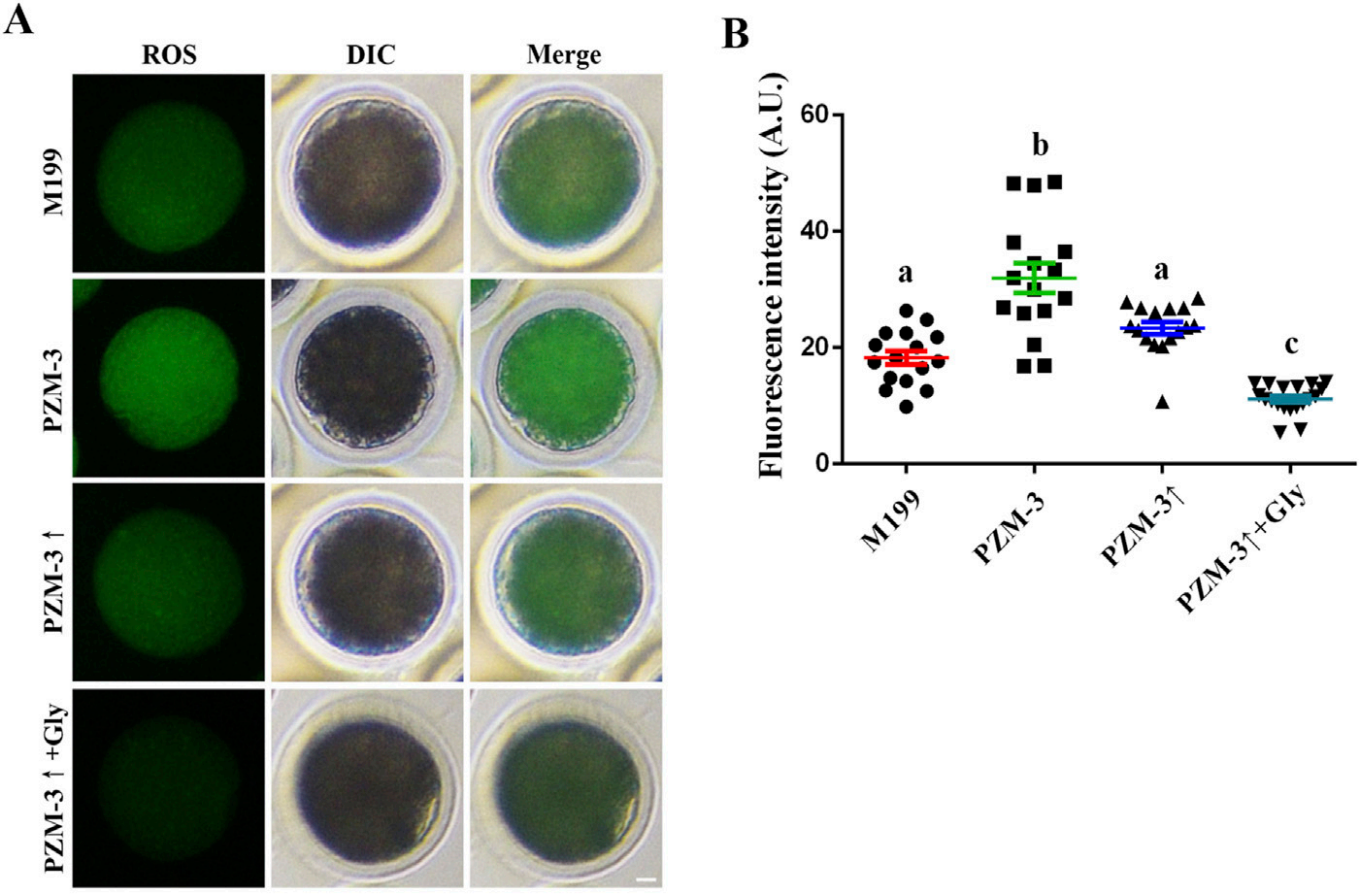
Effects of different culture mediums on ROS levels in porcine oocytes. **(A)** Immunofluorescent visualization of the ROS in oocytes across different groups, with ROS depicted in green (scale bar: 10 μm). The images include Differential Interference Contrast (DIC) microscopy to provide cellular context; the merged image represents the combination of green ROS fluorescence and DIC. **(B)** Quantitative assessment of ROS fluorescence intensity within oocytes, comparing the means in different groups. All experiments were performed independently at least three times. Data are presented as the mean ± SEM. Different low case letters indicate statistical differences at *P* < 0.05.

ROS are known to activate intracellular signaling pathways that facilitate cellular growth and metabolism (Agarwal et al., 2012). However, an overabundance of ROS can inflict damage upon cellular DNA, lipids, and proteins, precipitating oxidative stress and mitochondrial dysfunction (van der Reest et al., 2021). ROS levels in porcine oocytes after 44 h of culture were measured. The PZM-3 group exhibited the highest ROS levels (31.93 ± 9.87, n = 16), which were significantly higher than those of the other groups. A decrease in ROS levels was observed in the PZM-3↑ group (23.38 ± 4.14, n = 16), yet these levels were not significantly different from those of the M199 group (18.25 ± 4.55, n = 16). The PZM-3↑ + Gly group displayed the lowest ROS levels (11.16 ± 2.42, n = 19), which were significantly lower than those of the other groups (Figure 4). These results indicate that regulation culture medium osmolarity in the presence of Gly significantly attenuates oxidative stress-induced injury during the IVM of porcine oocytes.

### Osmolarity regulation and Gly addition in the culture medium upregulate glycolytic gene expression in cumulus cells

**FIGURE 5:**
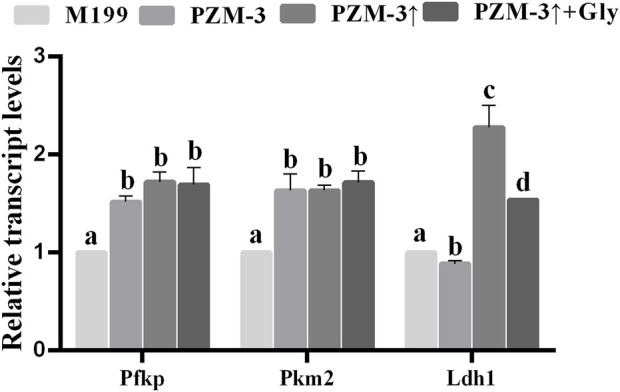
Effects of different culture mediums on the relative transcript abundance of glycolytic genes within CCs of porcine oocytes. A quantitative assessment of glycolytic gene expression was conducted, with transcript levels normalized against the endogenous control gene, GAPDH. All experiments were performed independently at least three times. Data are presented as the mean ± SEM. Different low case letters indicate statistical differences at *P* < 0.05.

One of the functions of cumulus cells (CCs) is to transport metabolites and nutrients to oocytes to help stimulate the rupture of germinal vesicles and improve the development of metaphase II (MII) (Xie et al., 2023). To elucidate whether the amelioration in porcine oocyte maturation quality within the PZM-3 medium, after the osmolarity adjustment in the presence of Gly, is associated with the bidirectional communication with the associated CCs, a comparative analysis of the transcriptional levels of key glycolytic genes—*Ldh1*, *Pkm2*, and *Pfkp*—was conducted on CCs derived from each experimental group. The transcriptional levels of *Pfkp* and *Pkm2* were significantly elevated in both the PZM-3↑ and PZM-3↑ + Gly groups compared to these in the M199 group. In contrast, the PZM-3 group exhibited a significant reduction in *Ldh1* transcriptional levels compared to the M199 group, whereas both the PZM-3↑ and PZM-3↑ + Gly groups displayed a marked increased level of *Ldh1* transcripts (Figure 5). These outcomes suggest that optimizing cell volume regulation during oocyte maturation substantially upregulate the expression of glycolytic genes in CCs.

### Optimizing the cell volume regulation of porcine oocytes during meiosis substantially enhance the quality of PA and IVF blastocysts

**FIGURE 6:**
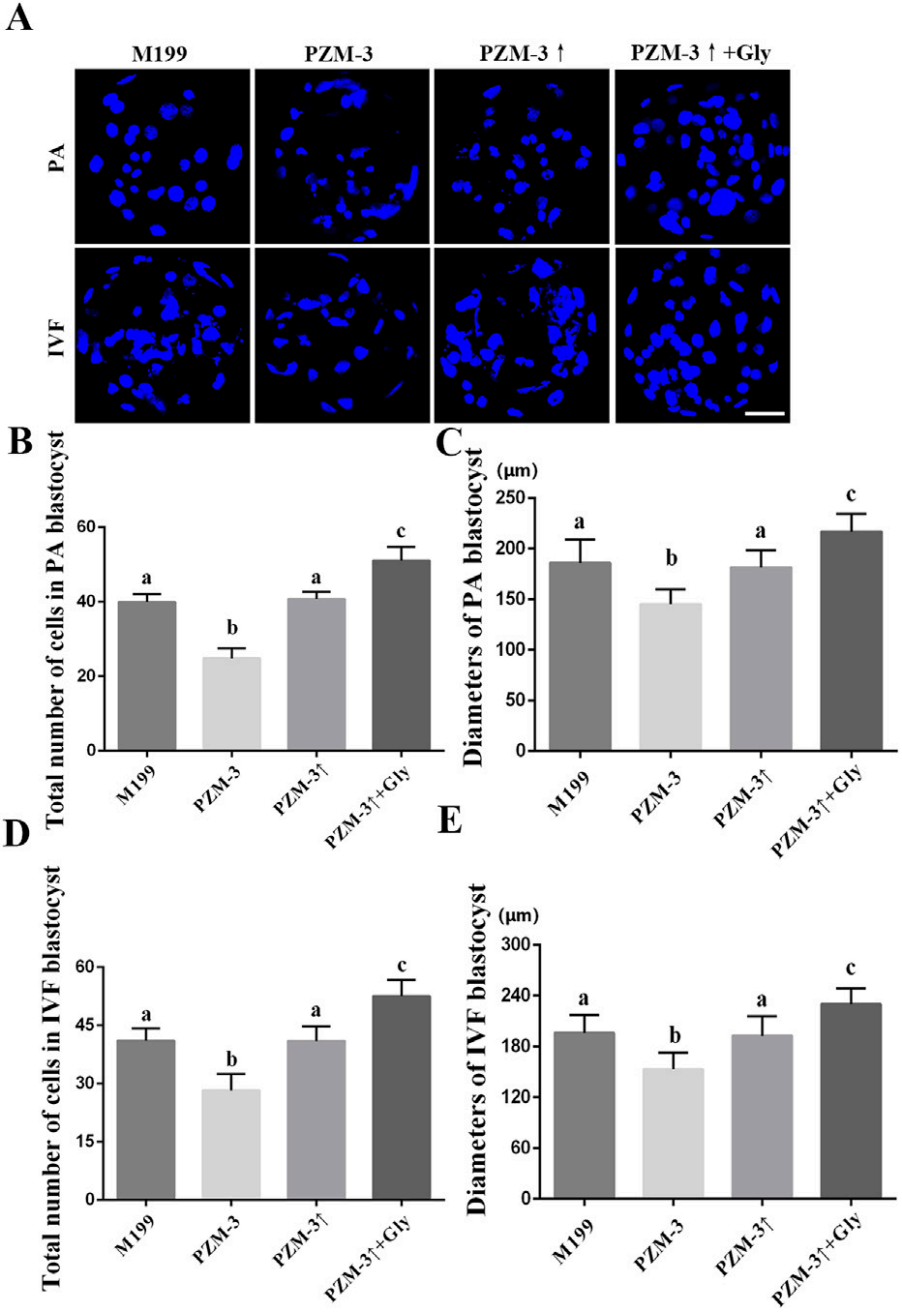
Effects of different culture mediums on the morphological quality of porcine blastocysts derived from PA and IVF. **(A)** Photomicrographs illustrating representative DNA staining of blastocysts from different treatment groups, with DAPI used for nuclear counterstaining (depicted in blue) (scale bar: 50 μm). **(B, D)** Quantification of the total cell count within blastocysts in different groups. **(C, E)** Measurement of blastocyst diameter in different groups. All experiments were performed independently at least three times. Data are presented as the mean ± SEM. Different low case letters indicate statistical differences at *P* < 0.05.

To assess the impact of the optimized maturation culture medium, on the developmental quality of blastocysts, matured oocytes from disparate groups were subjected to PA and IVF. The ensuing blastocysts underwent nuclear staining and diameter measurement. The blastocysts from the PZM-3 group, derived through PA, demonstrated impaired development, with a total cell count (24.88 ± 2.54, n = 16) and diameter (145.31 ± 14.19, n = 16) significantly lower than those of the M199 group (39.90 ± 2.05, n = 20 and 186.00 ± 22.28, n = 20, respectively). Following osmolarity optimization in the PZM-3↑ group, the blastocysts exhibited development comparable to the M199 group, with a total cell count (41.50 ± 2.44, n = 20) that was not significantly different. Furthermore, The total cell number (51.10 ± 3.53, n = 20) and diameter (217.00 ± 16.76, n = 20) of blastocysts from the PZM-3↑ + Gly group were significantly higher than those in the M199 group. The development of the IVF blastocysts in each group was consistent with the trend of the blastocysts from PA. The specific data are shown in Figure 6. These findings underscore that optimizing cell volume regulation during oocyte maturation significantly bolsters the developmental potential of oocytes, culminating in superior blastocyst quality.

## Discussion

The current results demonstrate optimizing cell volume regulation through stage-dependent osmolarity adjustment of simplified medium PZM-3 from 290 mOsM for the first 22 h into 320 mOsM significantly improved the quality of porcine oocyte maturation *in vitro*, manifested by the oocyte maturation rate, functional mitochondrial distribution and activity, the transcription level of glycolysis genes in CCs, and subsequent embryonic developmental ability and ROS levels in the presence of 1 mM Gly. We predicted that porcine oocytes can adapt to changes of osmolarity of culture medium during IVM and use Gly to maintain cell volume homeostasis, through protecting organelles and bi-directional communication between oocyte and its companion CCs from irreversible damage and lowering the oxidative stress.

The growth of the oocyte transpires within the confines of the zona pellucida, with the plasma membrane giving rise to microvilli that project into the zona as transzonal projections (TZPs) (Li and Albertini, 2013; Clarke, 2018). Upon initiation of meiosis or following *in vitro* culture post-detachment from the follicular microenvironment, TZPs undergo fragmentation (cleaved by a metallopeptidase), prompting the oocyte to autonomously regulate its cellular volume (Macaulay et al., 2014; Yuan et al., 2017; Macaulay et al., 2023). Our current results indicate that adjusting the osmolarity of PZM-3 culture medium from low to high (290 mOsM for the first 22 h and 320 mOsM thereafter) using raffinose significantly increased the rate of first polar body extrusion and subsequent cleavage rate of oocytes. This is consistent with the findings that osmolarity changes induced by NaCl or sucrose increased the cleavage rate of pig oocytes on day 2 (Miyoshi and Mizobe, 2014). Our research indicates that maintaining a 320 mOsM osmolarity of culture medium during the whole IVM or adjusting the osmolarity from high to low reduces the efficiency of oocyte maturation and cleavage rate. Studies have reported that the osmolarity of the culture medium within the range of 273–361 mOsM has a significant impact on the cleavage rate (Miyoshi and Mizobe, 2014). These results suggest that osmolarity of culture medium is a key factor affecting the porcine oocyte meiotic maturation, rather than raffinose itself, which cannot be used by animal cells.

During the growth and development of porcine follicles, half of the concentration of Gly in follicular fluid is consumed (Hong and Lee, 2007), suggesting that Gly may be an important factor promoting oocyte maturation. Studies have reported that Gly plays a crucial role in oocyte maturation and subsequent development by regulating ROS-induced lipid metabolism to prevent biological membrane damage (Gao et al., 2023). Our results indicate that adjusting the osmolarity of PZM-3 culture medium in the presence of 1 mM Gly has a more significant beneficial effect on the maturation of pig oocytes. This result confirms the theoretical proposition of Jennifer L. that the culture medium used to simulate *in vivo* conditions for oocyte maturation should include physiological osmotic pressure and organic osmolytes (Collins and Baltz, 1999). Mitochondria, organelles distinct in harboring their genetic material, undergo a substantial increase in mtDNA copy number and exhibit significant redistribution during oocyte maturation (Kirillova et al., 2021). Our results indicate that adjusting the osmolarity of PZM-3 culture medium from low to high (290 mOsM for the first 22 h and 320 mOsM thereafter) in the presence of 1 mM Gly enhances the distribution and activity of functional mitochondria within oocytes. Studies have reported that Gly can enhance mitochondrial function by regulating Ca^2+^, thereby promoting the oocyte maturation (Yu et al., 2021). During *in vitro* culture, as the osmolarity of the culture medium increases, the organic osmolyte Gly displaces some charged inorganic ions (Tscherner et al., 2021). These findings suggest that during the IVM of porcine oocytes, Gly improves cytoplasmic maturation by enhancing the distribution and activity of functional mitochondria.

Due to the lack of transporter of glucose uptake, the ability of oocytes to acquire glucose is limited (Sutton-McDowall et al., 2010). Therefore, oocytes rely on CCs to take up glucose on their behalf and metabolize it into pyruvate through glycolysis, which is then provided to the oocytes for metabolism via mitochondrial oxidative phosphorylation (OXPHOS) (Biggers et al., 1967; Gardner et al., 1996). Our study indicates that optimizing cell volume regulation through stage-dependent osmolarity adjustment of PZM-3 significantly increased the transcription level of glycolytic genes in CCs, providing a basis for the production of pyruvate through glycolysis in CCs, thereby supplying the necessary ATP for oocyte maturation. OXPHOS produces a large amount of ATP while also generating ROS as a byproduct. ROS are a double-edged sword in the development of oocytes, possessing important cellular signaling functions, but excessive accumulation can induce oxidative stress (Sies, 1997; Agarwal et al., 2012). Our research indicates that adjusting the osmolarity of PZM-3 culture medium from low to high in the presence of 1 mM Gly can effectively reduce the accumulation of intracellular ROS. Gly is an important component of the antioxidant glutathione (GSH) (Park et al., 2023). GSH is the most abundant intracellular antioxidant thiol and is at the core of redox defense during oxidative stress (Biswas and Rahman, 2009). These results suggest that in the later stages of oocyte maturation, Gly timely eliminates accumulated ROS through its antioxidant action, thereby reducing oxidative stress during oocyte maturation. Cortical granules (CGs) originate from the Golgi apparatus and play an important role in the preparation of oocytes for fertilization. The exocytosis of CGs post-fertilization prevents sperm penetration into the zona pellucida, thus averting polyspermy (Sun, 2003; Liu, 2011). Our study showed that the above adjustments to the PZM-3 medium improved the migration of CG (see Supplementary Figure 2). The quality of oocyte maturation is a decisive factor for embryonic development (Jiang et al., 2023). Our results indicate that the blastocysts from PA or IVF of oocytes matured in the optimized PZM-3 culture medium have higher quality with increased total cell number and diameter of blastocysts. Studies have reported that adding 6 mM Gly to the maturation media significantly increased the blastocyst rate after PA of pig oocytes and reduced apoptosis levels (Li et al., 2018). Hiroaki Funahashi et al. reported that the microfilament organization in pig oocytes was disrupted in maturation media containing higher levels of NaCl (92.40 mM). The addition of 12 mM sorbitol reduced the severity of abnormalities (Funahashi et al., 1996). Therefore, we speculate that the improvement in embryonic development capability and quality is due to the protection of oocyte microfilament organization provided by the optimized culture medium, thereby ensuring the mitotic capability of the embryo. Whether further optimization of embryonic culture media can improve embryonic development capability remains to be further studied.

In conclusion, optimizing cell volume regulation through stage-dependent osmolarity adjustment of medium from 290 mOsM for the first 22 h into 320 mOsM can effectively eliminate ROS accumulation, improve the distribution and activity of functional mitochondria, increase the transcription activity of glycolysis genes in CCs, enhance the migration of CG, and ultimately improve embryonic development potential.

## Data Availability

The original contributions presented in the study are included in the article/Supplementary Material, further inquiries can be directed to the corresponding author.
