# Supplementary material for: Stage-dependent changes in culture medium osmolality promote porcine oocyte maturation in vitro
Source: Front Cell Dev Biol. 2025 Jan 30;13:1524749. doi: 10.3389/fcell.2025.1524749 (PMC11821615; doi:10.3389/fcell.2025.1524749)
Supplement: Supplementary file 1 [file DataSheet1.docx]

***Supplementary Material***

1. **Supplementary Figures**


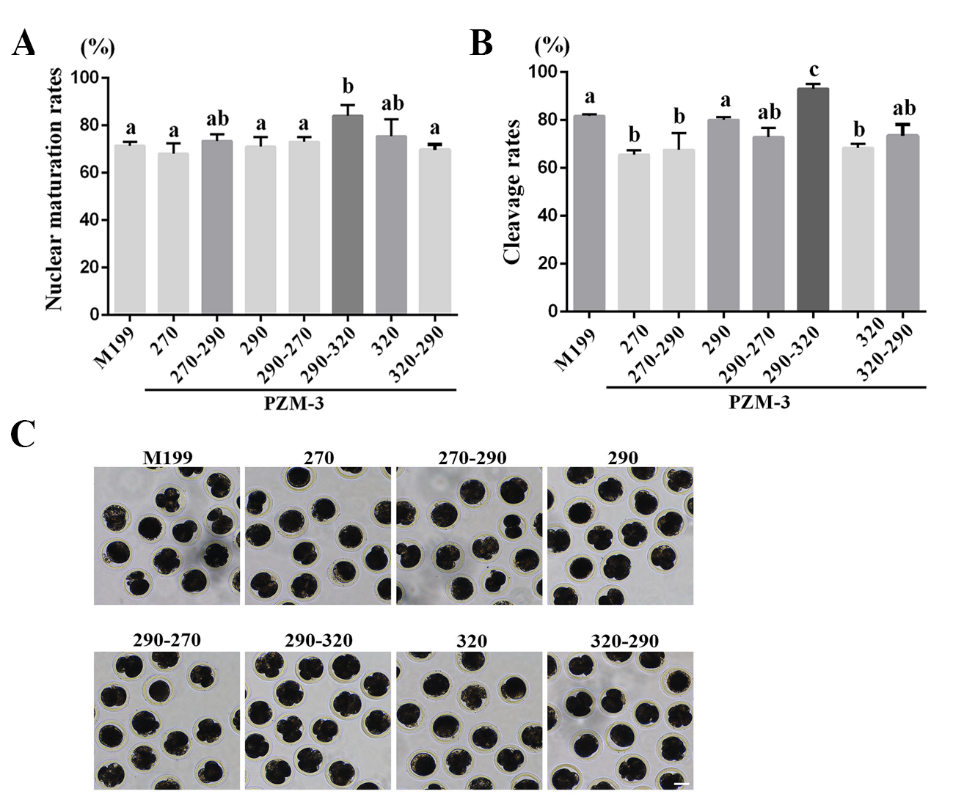


**Supplementary Figure 1.** Effects of different culture mediums on porcine oocyte maturation and PA cleavage rates. A. Nuclear maturation rate, represented by the percentage of oocytes with the first polar body in each group (number of MII oocytes/number of viable oocytes). B. Cleavage rate, indicated by the percentage of PA embryos with ≥2 cleavage balls in each group (number of cleaved embryos/number of PA embryos). C. Representative images of cleavage in PA embryos (scale bar: 50 μm). All experiments were performed independently at least three times. Data expressed in proportions were analyzed after arcsine transformation. Data are presented as the mean ±standard error of the mean (SEM). Different low case letters indicate statistical differences at *P*＜0.05.

M199: Oocytes were matured in a M199-based medium for 44 h in vitro.

270: Oocytes were matured in a PZM-3-based medium for 44 h in vitro. The osmolality of the medium was 270 mOsM.

270-290: Oocytes were matured in a PZM-3-based medium for 44 h in vitro. The osmolarity was initially adjusted at 270 mOsM for the first 22 h, followed by an adjustment to 290 mOsM for the remaining 22 h.

The osmolarity of groups 290, 290-270, 290-320, 320, and 320-290 was adjusted with reference to groups 270 and 270-290.


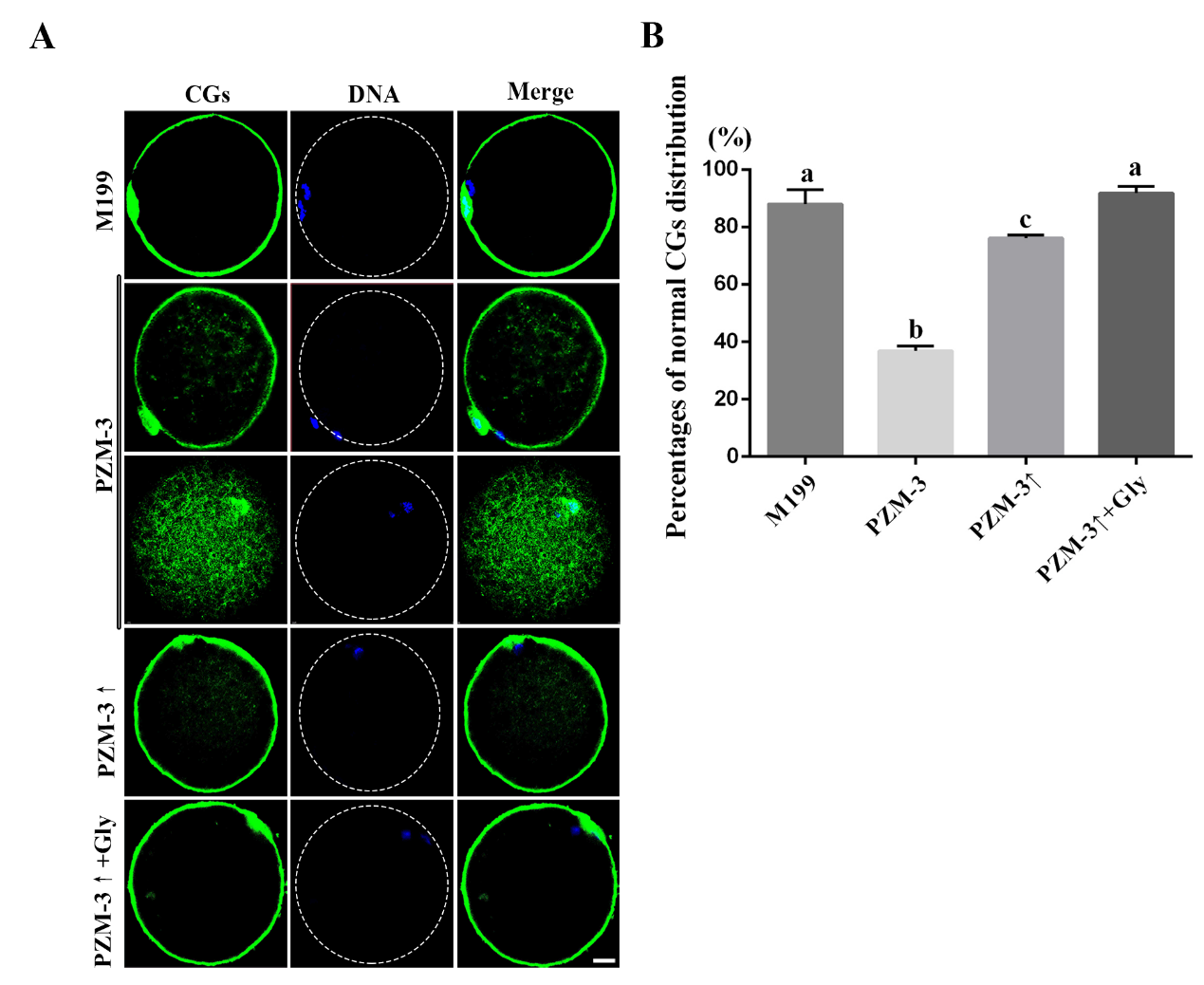


**Supplementary Figure 2.** Effects of different culture mediums on CGs migration in porcine oocytes during maturation. A. Immunofluorescent visualization of the distribution of CGs in oocytes, visualized by green fluorescence. DAPI nuclear staining is shown in blue; the merged image illustrates the superimposition of CGs fluorescence (green) and nuclear fluorescence (blue) (scale bar: 10 μm). B. Quantitative assessment of the proportion of oocytes exhibiting a normal distribution pattern of CGs within each experimental group. All experiments were performed independently at least three times. Data expressed in proportions were analyzed after arcsine transformation. Data are presented as the mean ±SEM. Different low case letters indicate statistical differences at *P*＜0.05.
